# Peptide-Bound Methionine Sulfoxide (MetO) Levels and MsrB2 Abundance Are Differentially Regulated during the Desiccation Phase in Contrasted *Acer* Seeds

**DOI:** 10.3390/antiox9050391

**Published:** 2020-05-07

**Authors:** Natalia Wojciechowska, Shirin Alipour, Ewelina Stolarska, Karolina Bilska, Pascal Rey, Ewa Marzena Kalemba

**Affiliations:** 1 Institute of Dendrology, Polish Academy of Sciences, Parkowa 5, 62-035 Kórnik, Poland; natalia.wojciechowska@amu.edu.pl (N.W.); salipour@man.poznan.pl (S.A.); ewelina.stolarska89@gmail.com (E.S.); mgr.karolina.bilska@gmail.com (K.B.); 2 Department of General Botany, Institute of Experimental Biology, Faculty of Biology, Adam Mickiewicz University, Uniwersytetu Poznańskiego 6, 61-614 Poznań, Poland; 3 Department of Forestry, Faculty of Agriculture and Natural Resources, Lorestan University, Khorramabad, Iran; 4 Aix Marseille University (AMU), Commissariat à l’Energie Atomique et aux Energies Alternatives (CEA), Centre National de la Recherche Scientifique (CNRS), Biosciences and Biotechnology Institute of Aix-Marseille (BIAM), Plant Protective Proteins (PPV) Team, 13108 Saint Paul-Lez-Durance, France; pascal.rey@cea.fr

**Keywords:** desiccation, methionine sulfoxide, methionine sulfoxide reductases, Norway maple, reactive oxygen species, seeds, sycamore

## Abstract

Norway maple and sycamore produce desiccation-tolerant (orthodox) and desiccation-sensitive (recalcitrant) seeds, respectively. Drying affects reduction and oxidation (redox) status in seeds. Oxidation of methionine to methionine sulfoxide (MetO) and reduction via methionine sulfoxide reductases (Msrs) have never been investigated in relation to seed desiccation tolerance. MetO levels and the abundance of Msrs were investigated in relation to levels of reactive oxygen species (ROS) such as hydrogen peroxide, superoxide anion radical and hydroxyl radical (•OH), and the levels of ascorbate and glutathione redox couples in gradually dried seeds. Peptide-bound MetO levels were positively correlated with ROS concentrations in the orthodox seeds. In particular, •OH affected MetO levels as well as the abundance of MsrB2 solely in the embryonic axes of Norway maple seeds. In this species, MsrB2 was present in oxidized and reduced forms, and the latter was favored by reduced glutathione and ascorbic acid. In contrast, sycamore seeds accumulated higher ROS levels. Additionally, MsrB2 was oxidized in sycamore throughout dehydration. In this context, the three elements •OH level, MetO content and MsrB2 abundance, linked together uniquely to Norway maple seeds, might be considered important players of the redox network associated with desiccation tolerance.

## 1. Introduction

Desiccation is a phase between seed maturation and germination and is associated with a major loss of water in preparation of the dormancy period and, later, of the germination stage [1,2]. Based on their desiccation tolerance, seeds are classified as either orthodox (resistant) or recalcitrant (sensitive) [3]. Orthodox seeds survive extreme dehydration without a loss in viability; however, the conservation and propagation of recalcitrant seeds is much more difficult [4,5,6]. Desiccation tolerance is an essential adaptation that enabled the colonization of terrestrial habitats by early land plants; however, this adaptation was lost in some plants growing in environments where recalcitrance supports rapid germination [7]. Desiccation tolerance is regulated by mechanisms related to the activation of protective mechanisms [2,8], and these include entry into a quiescent state; the filling of vacuoles [9,10]; the accumulation of protective molecules such as sugars [9,11], late embryogenesis abundant proteins [12], and heat shock proteins [13,14]; and the production of antioxidants assumed to be fundamental in desiccation tolerance [15,16]. Protective molecules are extensively produced in response to water shortage that occurs during maturation drying [17], and this phenomenon is unique to orthodox seeds [5,18]. Protective mechanisms further increase seed longevity [19], which differs between orthodox and recalcitrant seeds [20].

Desiccation of seeds leads to an imbalance in metabolism and causes intracellular damage and loss of seed viability within weeks or months depending on the species [21]. These consequences of desiccation are related to the accumulation of reactive oxygen species (ROS), among other factors. ROS are dynamically generated in plants [22]; however, they play a dual role in seeds [23], as they are an element of signaling pathways in seeds [24] and induce oxidative stress that damages DNA, proteins and lipids and eventually causes cell death [25]. Excessive accumulation of ROS such as hydrogen peroxide (H_2_O_2_), superoxide radical (O_2_•^−^), and hydroxyl radicals (•OH) cause triphasic seed deterioration whose symptoms are observed in both recalcitrant seeds and orthodox seeds [26]. ROS are also involved in the oxidation of amino acids such as cysteine or methionine (Met) and thus participate in reversible posttranslational modifications [27]. In particular, Met residues are susceptible to combining with ROS and are then converted to methionine sulfoxide (MetO). Among ROS, •OH has the highest oxidative potential in the Met to MetO transformation [28]. MetO is reduced back to Met by the methionine sulfoxide reductase (Msr) system, which consists of MsrA and MsrB isoforms [29,30,31,32]. MsrA and MsrB enzymes display distinct specificity on MetO diastereoimers. MsrA can reduce both a free and protein-bound S-diastereomer of MetO, whereas MsrB is specific for the protein-bound R-diastereomer [33]. Msrs play protective roles in plants under stress conditions (reviewed in [34]). Based on the cis-regulatory elements within the promoters of all Msr genes from Arabidopsis, poplar and rice [27,29,35], Msrs are presumed to regulate many aspects of plant life [36]. A type Mrs was found to regulate Arabidopsis growth in short-day conditions [37] and to be involved in dormancy induction [38]. Among the Arabidopsis Msrs, two—AtMsrB2 and AtMsrB6—have been identified in seeds [39]. The accumulation of MsrB is related to the reestablishment of desiccation tolerance in germinating seeds [40]. MsrB1 and MsrB2 were recently found in developing Norway maple and sycamore seeds [41]. The dissimilar abundance of the two proteins was assumed to be related to the acquisition of desiccation tolerance [41], which is unique to Norway maple seeds [42]. Recalcitrant seeds, in contrast to orthodox ones, are difficult to store due to rapid viability loss [5,18]. Interestingly, Châtelain et al. [39] established that the Msr repair system plays a decisive role in establishing and preserving seed longevity. Therefore, we were encouraged to determine the involvement of the two MsrB isoforms in the response to dehydration and desiccation in seeds with contrasting physiology in terms of desiccation tolerance.

Two *Acer* species, Norway maple (*Acer platanoides* L.) and sycamore (*Acer pseudoplatanus* L.), that grow under similar conditions in the temperate zone produce orthodox pollen [43] but also orthodox and recalcitrant seeds, respectively [44,45], and the seeds of both species are characterized as undergoing deep physiological dormancy [46]. These two species are, therefore, excellent models for studying the differences enabling the production of seeds with contrasting physiology during development [41,42,47], dormancy [38,48], and drying and desiccation [44,47,49,50,51]. In this work, we focused on the response to the gradual drying in these two opposite seed types: orthodox (Norway maple) and recalcitrant (sycamore). Principally, we investigated the levels of peptide-bound MetO and abundance of MsrB proteins. Peptide methionine sulfoxide reductase A was found to accumulate in developing Norway maple seeds, but was considered a protein involved in dormancy induction [38]. To elucidate whether Msrs could play a role in desiccation tolerance we focused on MsrB1 and MsrB2 which have been already characterized as essential during seed development [41] and for seed longevity [39]. The role of MetO and integration of MsrBs in the redox network controlling seed physiology in contrasted *Acer* seeds was supported by established relationships of these parameters with ROS levels and elements of the ascorbate-glutathione cycle.

## 2. Materials and Methods

### 2.1. Seed Material

Mature seeds were collected from two species of *Acer*: Norway maple and sycamore. The seeds were collected from individual trees growing in Kórnik Arboretum, Western Poland, 52°24’37’’ N, 17°09’515’’ E, at 23 and 24 weeks after flowering. Water content (WC) was measured in triplicate in ten embryonic axes and five cotyledons dried at 105 °C for 24 h. The seeds were dehydrated at ambient temperature (20 °C) and 60% relative humidity. The Norway maple seeds were desiccated to 7–10% WC, while the sycamore seeds were dried to 30% WC. Samples were collected at intervals of 10% WC. The seed coat was removed prior to weighing and frozen at −80 °C. Twenty embryonic axes and five cotyledons were taken per sample for analysis.

### 2.2. Determination of ROS Levels

#### 2.2.1. Determination of H_2_O_2_ Release

The release of H_2_O_2_ was determined according to the method described by Schopfer et al. [52]. Six replicates of four whole seeds were incubated in 1.2 mL of a reaction mixture composed of a 20 mM phosphate buffer (pH 6), 5 μM scopoletin and 1 U mL^−1^ peroxidase. The samples were incubated in darkness on a shaker at 150 rpm for 1 h at room temperature (RT). The supernatant was subsequently clarified by short centrifugation, after which the fluorescence was measured at an excitation wavelength of 346 nm and an emission wavelength of 455 nm using an Infinite M200 PRO (Tecan, Männedorf, Switzerland) plate reader and Magellan software. The results are expressed in picomoles of H_2_O_2_ per gram of dry weight (DW) per hour.

#### 2.2.2. Determination of O_2_•^−^ Release

The level of O_2_•^−^ was determined by adopting the method of Choi et al. [53]. Seed samples were incubated for 30 min at RT in the dark in 1.2 mL of a reaction mixture consisting of a 50 mM phosphate buffer (pH 7.8), 0.05% nitro blue tetrazolium (NBT; Sigma, St. Louis, MO, USA), and 10 mM sodium azide. Afterward, 750 μL of the solution was transferred to dark tubes, heated for 30 min at 85 °C and then cooled on ice. Seventy-five microliters of the solution was then centrifuged for 90 s at 10,000× *g*. The precipitate was dissolved in dimethyl sulfoxide (DMSO) consisting of 2 M KOH by shaking for 30 min at 150 rpm and vortexing every 5 min. Samples diluted in DMSO were measured at 719 nm using an Infinite M200 PRO (Tecan, Männedorf, Switzerland) plate reader and Magellan software. The results are expressed in ΔA_719_ values per gram of DW per hour.

#### 2.2.3. Determination of •OH Release

The release of •OH was determined according to the methods of Schopfer et al. [52]. Six replicates of four whole seeds were incubated in 1.2 mL of a reaction mixture consisting of a 20 mM phosphate buffer (pH 6) and 2.5 mM sodium benzoate. Samples were incubated on a shaker at 150 rpm for 3 h at RT in darkness. The supernatant was then clarified by short centrifugation. The fluorescence was measured at an excitation wavelength of 305 nm and an emission wavelength of 407 nm using an Infinite M200 PRO (Tecan, Männedorf, Switzerland) plate reader and Magellan software. The results were expressed in relative fluorescence units (RFU) per gram of DW per hour.

#### 2.2.4. Histochemical Detection of ROS

H_2_O_2_ was visualized in whole Norway maple seeds and sycamore seeds as a reddish-brown stain formed by the reaction with 1 mg mL^−1^ 3,3′-diaminobenzidine (DAB; Sigma, St. Louis, MO, USA) solution in sodium phosphate buffer according to the method of Daudi and O’Brien [54] with several modifications [55]. O_2_•^−^ was visualized as a dark blue-to-black stain formed by NBT using 0.2% NBT solution in sodium phosphate buffer (pH 7.5) according to the protocol of Kumar et al. [56]. Images were taken on a plain white background using a Nikon D3100 digital camera attached to a binocular microscope.

### 2.3. Determination of Peptide-Bound MetO Level

Determination of MetO, Met, tyrosine (Tyr), and tryptophan (Trp) were performed according to the method described by Baxter et al. [57] using an Agilent Infinity II 1260 model HPLC system (Agilent Technologies, Wilmington, DE, USA) equipped with an Agilent Poroshell 120 StableBond-Aq (3.0 × 150 mm, 2.7 µm) particle column heated to 40 °C and mobile phases based on water (A) and potassium phosphate buffer combined with acetonitrile and isopropanol (B). A sample containing 2.8 mg of proteins isolated in PIPES buffer (pH 7.5) was digested (20 h/37 °C) using a combination of three enzymes: pronase, leucine aminopeptidase, and prolidase. The detection wavelengths were 214 (for MetO and Met) and 280 nm (for Tyr and Trp), with references at 590 nm. DL-methionine sulfoxide (∼99%) was used as a standard, thus MetO refers to a pool of both enantiomers. The only modifications to the method were adjustments to the model of chromatography and the column. Thus, the elution program was 0% B from 0.0 to 5.0 min (flow rate of 0.15 mL min^−1^), 0 to 16% B from 5.0 to 8.0 min (flow rate of 0.3 mL min^−1^), 16 to 100% B from 8.0 to 16.0 min (flow rate of 0.3 mL min^−1^), and 0% B from 16.0 to 18.0 min (flow rate from 0.3 to 0.15 mL min^−1^). The MetO ratio was calculated in relation to the total pool of Met detected.

### 2.4. Protein Extraction

The embryonic axes and cotyledons were ground in liquid nitrogen in a chilled mortar and pestle. The dry powder was incubated in extraction buffer composed of Tris-Cl, glycerol, and β-mercaptoethanol together with polyvinylpolypyrrolidone at 4 °C for one hour, with shaking every 15 min. The homogenates were centrifuged for 20 min at 20,000× *g* at 4 °C. The protein concentration in the collected supernatant was subsequently measured using the Bradford method [58].

### 2.5. Western Blot Analysis

Proteins were separated by SDS-PAGE on 12–17% polyacrylamide gels, with an equal amount of protein (20 μg) in each lane. Transfer to the polyvinylidene fluoride (PVDF) membrane was performed using Trans-Blot^®^ Turbo™ (Bio-Rad, CA, USA). The PVDF membrane was blocked in 5% skimmed milk dissolved in phosphate-buffered saline (PBS) at pH 7.4 for 1 h at RT. The primary antibodies against AtMsrB1 and AtMrB2 [59] were diluted 1:1000 in 5% skimmed milk dissolved in PBS. The incubation with primary antibodies was performed overnight at 4 °C. Secondary antibodies conjugated with horseradish peroxidase (HRP, Agrisera, Sweden) were diluted 1:10,000 in 5% skimmed milk dissolved in PBS. The PVDF membrane was incubated with Clarity Western ECL substrate chemiluminescent detection reagent (Bio-Rad, CA, USA) for 5 min prior to image registration in a G:BOX Chemi XR5 instrument (Syngene, Cambridge, UK). In addition to the Western blot (WB) technique described above, revelations were also performed using an alkaline phosphatase (AP)-labeled antibody. This less sensitive method allowed the detection of two close MsrB2 bands having distinct redox states. Secondary antibodies conjugated with alkaline phosphatase (AP, Sigma-Aldrich, St. Louis, MO, USA) were diluted 1:4000 in 5% skimmed milk. Proteins were detected colorimetrically using 5-bromo-4-chloro-3-indolyl phosphate (Sigma, St. Louis, MO, USA) and NBT (Sigma, St. Louis, MO, USA) as the AP substrate. Protein samples from the embryonic axes and cotyledons of both species were run on one gel and transferred on the same membrane to ensure identical detection conditions. WB images were analyzed densitometrically in triplicate using the UviBand (UviTec, Cambridge, UK) program of the Fire Reader Gel Documentation System. The band density was calculated based on the volume (V) of the band as the sum of all 3D intensities (I) coded on a scale of 256 gray levels. The data are presented in relative units obtained from V = Σn_i_I and the number of pixels inside the area of the band.

The two MsrB2 bands were examined to determine whether their different redox states are affected by AsA and GSH. We incubated 75 µg of the embryonic axes protein extract with AsA, as well as GSH (final concentration of 1 mM), for 30 min at RT. As a control, we omitted the step with AsA and GSH incubation.

### 2.6. Determination of Ascorbate and Glutathione Contents

The Asc and glutathione levels were measured according to the methods described by Queval and Noctor [60]. Seed samples were ground in 1 mL of 0.2 M HCl and centrifuged for 10 min at 10,000× *g* at 4 °C. The extract was neutralized to pH 4.5–5. An Infinite M200 PRO (Tecan, Männedorf, Switzerland) plate reader and Magellan software were used for all measurements.

The Asc assay was adapted from the methods of Hewitt and Dickes [61] and Queval and Noctor [60] and used by Stolarska et al. [41] to quantify extremely low levels of ascorbic acid (AsA) in dry seeds. AsA was measured in neutralized extracts on the basis of its ability to absorb light at 265 nm in a slightly acidic environment [61]. Asc, referred to as “total ascorbate”, was measured after conversion of dehydroascorbate (DHA) to AsA by incubation in 25 mM dithiothreitol at pH 4.7 [60]. The measurements were performed in 0.1-mM acetic acetate buffer consisting of 5 mM EDTA. DHA in the assays was determined by subtracting the level of free AsA from the total Asc.

To measure the two redox forms of glutathione, half of the neutralized extract was treated with 2 μL of 2-vinylpyridine (2-VP) for 30 min at RT and centrifuged twice at 14,000× *g* for 15 min at 4 °C, while the other half was untreated. The reaction mixture consisted of 120 mM NaH_2_PO_4_/10 mM EDTA (pH 7.5), 12 mM 5,5′-dithio-bis (2-nitrobenzoic acid) (DTNB), 10 mM NADPH, water, and extract (to measure the total glutathione, GSH + oxidized glutathione (GSSG)), or water plus 2-VP-treated extract (to measure GSSG), as well as glutathione reductase (0.2 U). The kinetic measurements were detected at 412 nm. The calculations were based on calibration curves prepared using GSSG and GSH (Sigma, St. Louis, MO, USA) as standards, and the degree of oxidation (DO) of glutathione was calculated according to the formula DO = (2[GSSG]/[GSH] + 2[GSSG]) × 100 described in Meyer and Hell [62].

### 2.7. Statistical Analyses

The data are the means of three independent replicates ± the standard deviations (STDs) or standard errors (SEs). Statistically significant differences are indicated with different letters (one-way ANOVA, followed by Tukey’s test at *p* ≤ 0.05). The relationships between particular parameters were evaluated using Pearson’s correlation coefficient analysis. Proportional data were transformed prior to analysis using the arcsine transformation. R statistical software was used to calculate Pearson’s correlation coefficients [63]. Correlation matrices were constructed using the corrplot package [64].

## 3. Results

Analyzed *Acer* seeds differed in initial and final levels of hydration. Mature Norway maple and sycamore seeds displayed 50% and 60% WC, respectively. Seed drying was conducted in 10% intervals in both species to limits enabling their viability. More specifically, Norway maple seeds were desiccated up to 10% WC, whereas sycamore seeds were dehydrated up to 30% WC. Thus, the three dehydration stages, namely 50%, 40%, and 30% WC, were overlapping.

### 3.1. ROS Contents in Acer Drying Seeds

**Figure 1:**
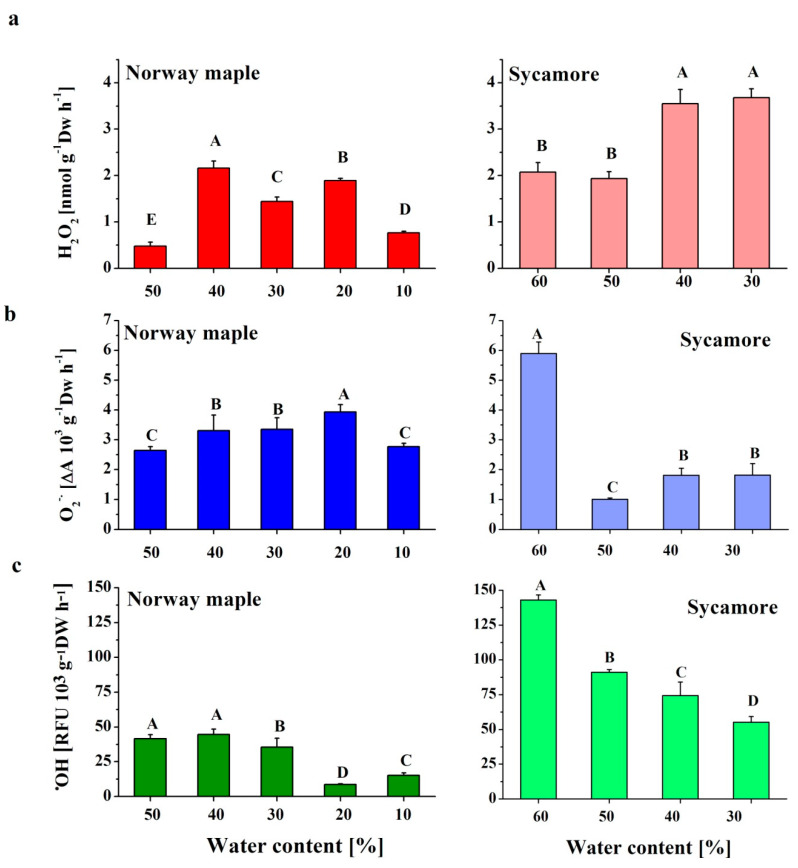
Detection of the ROS levels (**a**) hydrogen peroxide (H_2_O_2_), (**b**) superoxide anion radical (O_2_•^−^) and (**c**) hydroxyl radical (•OH) in Norway maple seeds desiccated to 10% WC and in sycamore seeds dehydrated to 30% WC. The data are the means of six independent replicates ± the STDs. The same letters indicate groups that are not significantly different according to Tukey’s test.

In the seeds of both *Acer* species, dehydration from 50 to 40% WC caused the highest increase in H_2_O_2_ production, with levels that nearly doubled in the sycamore seeds (Figure 1A). After this peak, the Norway maple seeds contained lower amounts of H_2_O_2_ upon progressive desiccation, whereas the amount of H_2_O_2_ continuously increased in sycamore seeds. When the seeds of the two species at the 30% WC dehydration stage were compared, the sycamore seeds exhibited twice as much H_2_O_2_ as the Norway maple seeds did.

In Norway maple seeds, the levels of O_2_•^−^ were slightly elevated upon desiccation to 20% WC but then decreased to their initial levels. The sycamore seeds exhibited different patterns of O_2_•^−^ content. Mature hydrated seeds contained much higher amounts of O_2_•^−^ as compared to other stages. Interestingly, dehydration to 50% WC resulted in a fivefold decrease in O_2_•^−^ levels. Further dehydration led to a somewhat increased O_2_•^−^ content to identical levels within the 40–30% WC range (Figure 1B).

Throughout all the dehydration stages, the sycamore seeds were characterized by definitively higher levels of •OH than the Norway maple seeds; however, in both species, a decreasing trend was observed (Figure 1). Fourfold decreases in •OH levels were detected at the 20% WC desiccation stage in the Norway maple seeds. Importantly, further desiccation doubled •OH content, but the average •OH levels in dry seeds were considerably lower than those detected at the 50–30% WC range. In the sycamore seeds, the most marked decrease in •OH levels was detected after dehydration to 50% WC. Afterward, the decrease was not as severe (Figure 1C). At the beginning of desiccation, ROS levels were much higher in sycamore than in Norway maple seeds. The higher ROS levels, except the O_2_•^−^, were still observed in sycamore seeds at the end of the dehydration phase.

The levels of two types of ROS, H_2_O_2_ and O_2_•^−^, was also monitored in whole seeds by using specific dyes, which displayed visible precipitants at the site of ROS origination (Figure S1). Both H_2_O_2_ and O_2_•^−^ were visualized in the embryonic axes and cotyledons of the seeds of both *Acer* species. The changes in the intensity of the precipitated dye reflected the precise measurements documented in Figure 1.

### 3.2. MetO Levels in Drying Acer Seeds

**Figure 2:**
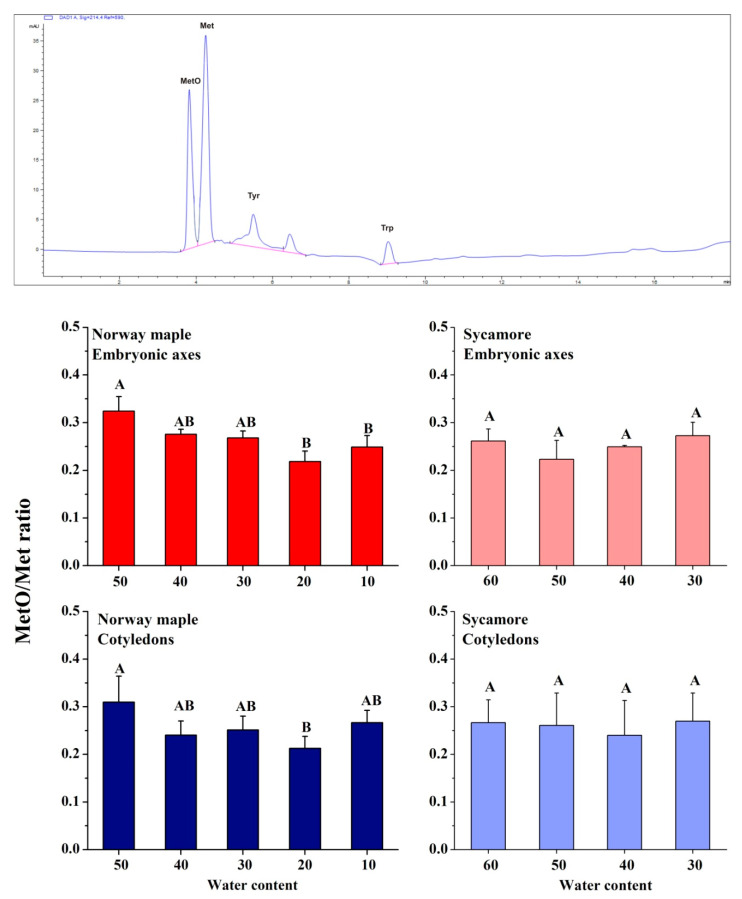
Proportion of Met oxidized to methionine sulfoxide (MetO) in proteins of desiccated Norway maple and dehydrated sycamore seeds. A representative chromatogram indicating peaks recognized at 214 nm is shown. Standards of Met, MetO, tyrosine (Tyr) and tryptophan (Trp) were used for the calibration curves. The MetO ratio was calculated in relation to the total pool of Met. The data are the means of three independent replicates ± the STDs. The same letters indicate groups that are not significantly different according to Tukey’s test.

The level of peptide-bound MetO is a hallmark of protein oxidation. At the beginning of seed drying, a MetO ratio higher by *ca* 5% was measured in Norway maple seeds than in sycamore. The levels of MetO changed upon desiccation solely in Norway maple seeds (Figure 2) and the ratios were similar at 30% WC stage in both species. Importantly, a decreasing trend was further detected in the Norway maple seeds along the desiccation phase. In the embryonic axes and cotyledons, the lowest MetO levels were recorded at the 20% desiccation stage. Dehydration had no effect on sycamore seeds in terms of methionine oxidation, no difference in MetO levels were noticed between 60% and 30% WC stages. At final stages of desiccation, the MetO levels in Norway maple embryonic axes decreased approximately by 10%.

### 3.3. MsrB1 and MsrB2 Content in Drying Acer Seeds

**Figure 3:**
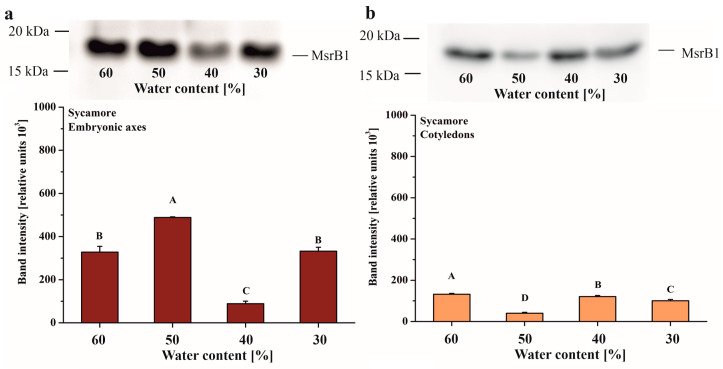
Immunoblot analyses and densitometric analysis of MsrB1 protein in the embryonic axes (**a**) and cotyledons (**b**) of sycamore. The data are the means of three independent replicates ± the STDs. The same letters indicate groups that are not significantly different according to Tukey’s test.

Immunodetection of the two B-type isoforms of Msrs, MsrB1 and MsrB2, was performed during desiccation of *Acer* seeds. The MsrB1 protein was not detectable in Norway maple seeds. In sycamore, MsBr1 was present from 60 to 30% WC, with the highest amount detected in the embryonic axes dehydrated to 50% WC (Figure 3A). MsrB1 was found in sycamore cotyledons at all stages of dehydration and, at the 50% WC dehydration stage, the abundance of this protein was lower compared to other stages (Figure 3B).

**Figure 4:**
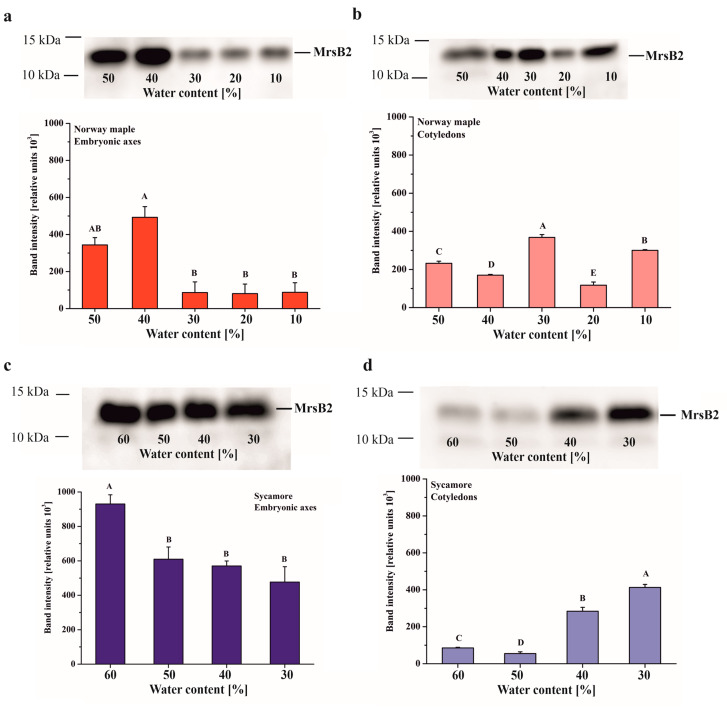
Immunoblot analyses and densitometric analysis of MsrB2 proteins in the embryonic axes (**a**, **c**) and cotyledons (**b**, **d**) of Norway maple (**a**, **b**) and sycamore (**c**, **d**). The data are the means of three independent replicates ± the STDs. The same letters indicate groups that are not significantly different according to Tukey’s test.

The MsrB2 protein was detected in seeds of both *Acer* species in embryonic axes as well as in cotyledons (Figure 4). The largest amount of this protein was revealed in Norway maple embryonic axes desiccated to 50% WC and especially 40%. Further desiccation (30–10% WC) caused a significant decrease in MsrB2 abundance, and this protein was barely detected (Figure 4A). The Norway maple cotyledons exhibited a different pattern since MsrB2 was detected at all desiccation stages, and the highest amounts were detected at 40%, 30% and 10% WC (Figure 4B).

MsrB2 was present both in the embryonic axes and in the cotyledons of sycamore seeds (Figure 4C,D). In the embryonic axis, the highest level of MsrB2 was observed in mature seeds (60% WC), while during desiccation (50–30% WC), a slight gradual decrease was observed (Figure 4C). The opposite trend was observed in cotyledons in which the amount of protein increased at final stages of the dehydration process, and the highest levels were reported in cotyledons dehydrated to 30% WC (Figure 4D).

### 3.4. Glutathione

**Figure 5:**
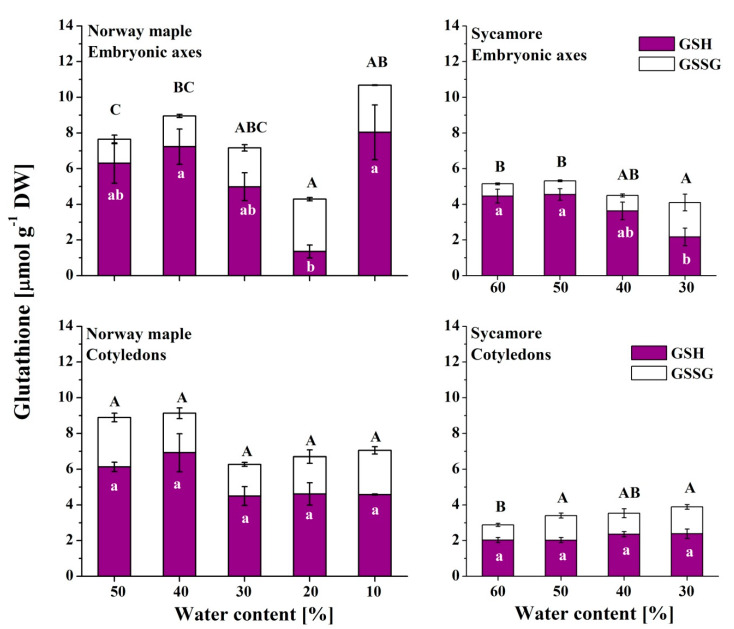
Levels of the reduced (GSH) and oxidized (GSSG) forms of glutathione measured in the embryonic axes and cotyledons of desiccated Norway maple and dehydrated sycamore seeds. The data are the means of three independent replicates ± the STDs. The same letters indicate groups that are not significantly different according to Tukey’s test. The capital letters refer to GSSG.

The reduced form of glutathione was predominant in Norway maple embryonic axes except at the 20% WC desiccation stage, when GSH levels decreased dramatically (Figure 5). Interestingly, further desiccation resulted in sixfold-increased levels of GSH, while the levels of GSSG remained stable. Globally, GSSG levels continuously increased in Norway maple embryonic axes while desiccation progressed. However, the levels of both glutathione forms were stable in Norway maple cotyledons throughout the desiccation process. In the sycamore cotyledons, the levels of glutathione were half as high as those of the Norway maple seeds. In the recalcitrant species, a decrease in GSH levels was detected in the embryonic axes during the final dehydration stages, and at the same time, increased GSSG levels were detected. Relatively stable levels of GSH were detected in progressively dehydrated sycamore cotyledons, whereas GSSG levels were higher in the 50–30% WC range than at the 60% WC stage.

**Figure 6:**
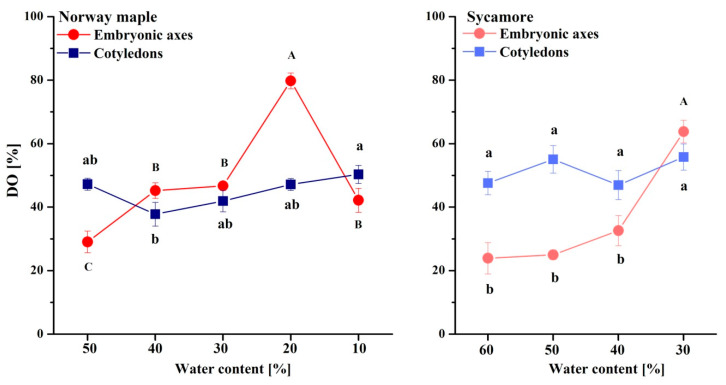
Degree of oxidation (DO) of glutathione in the embryonic axes and cotyledons of desiccated Norway maple and dehydrated sycamore seeds. The data are the means of three independent replicates ± the STDs. The same letters indicate groups that are not significantly different according to Tukey’s test. The capital letters refer to cotyledons.

The DO of glutathione clearly increased in the Norway maple embryonic axes and peaked at 20% WC desiccation stage (Figure 6). Further desiccation halved the DO. In the cotyledons, the DO was relatively constant within the 40–50% range. The DO in the embryonic axes of the sycamore seeds was relatively low and stable until dehydration to 40% WC, after which the DO doubled. The DO fluctuated by approximately 50% during all dehydration stages in the sycamore cotyledons. Interestingly, compared with embryonic axes, the DO in the cotyledons was twofold higher at the beginning of the dehydration of the sycamore seeds. Further dehydration resulted in an equal DO in whole sycamore seeds.

### 3.5. Ascorbate

**Figure 7:**
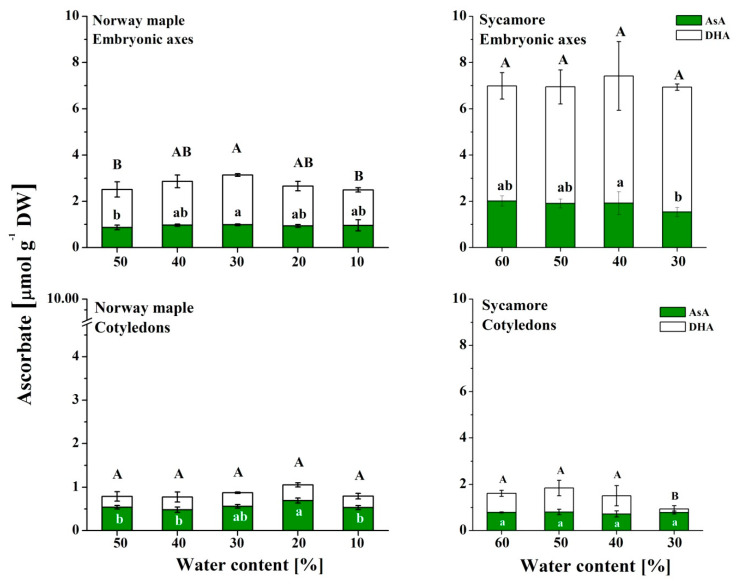
Levels of the reduced (AsA) and oxidized (DHA) forms of Asc reported in the embryonic axes and cotyledons of desiccated Norway maple and dehydrated sycamore seeds. The data are the means of three independent replicates ± the STDs. The same letters indicate groups that are not significantly different according to Tukey’s test. The capital letters refer to DHA.

Asc levels were higher in sycamore seeds than in Norway maple seeds (Figure 7). Tripled and doubled Asc levels were detected in the embryonic axes and cotyledons, respectively. During desiccation of the Norway maple embryonic axes, the levels of both AsA and DHA increased up to 30% WC, but then returned to their initial levels. The Norway maple cotyledons displayed Asc levels that were twofold lower than those of the embryonic axes. The AsA level was the highest in cotyledons at the 20% WC desiccation stage, whereas DHA levels were not affected at this stage. DHA was predominant in sycamore embryonic axes, and its levels did not change during the dehydration process; AsA levels were twofold lower throughout this process. In sycamore, the Asc levels were threefold lower in the cotyledons than in the embryonic axes. The AsA levels in the cotyledons did not change, but the DHA levels decreased substantially at the 30% WC dehydration stage.

**Figure 8:**
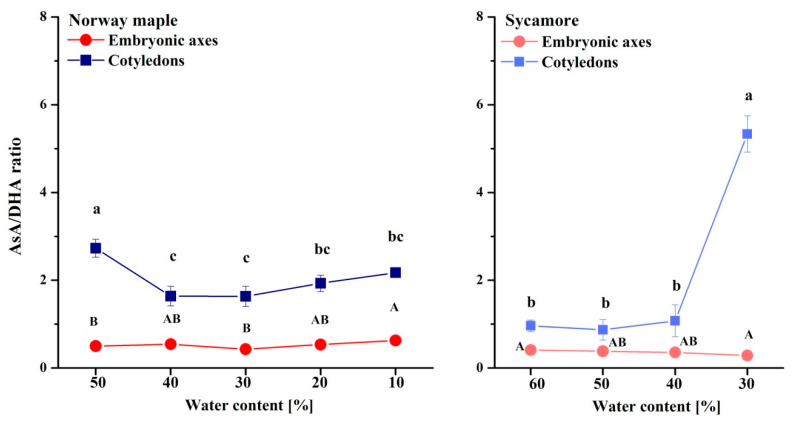
Ratios of reduced (AsA) to oxidized (DHA) forms of Asc in the embryonic axes and cotyledons of desiccated Norway maple and dehydrated sycamore seeds. The data are the means of three independent replicates ± the STDs. The same letters indicate groups that are not significantly different according to Tukey’s test. The capital letters refer to embryonic axes.

The AsA/DHA ratio was extremely low in the embryonic axes of the seeds of both *Acer* species as compared to cotyledons (Figure 8). The AsA/DHA ratio fluctuated around two in the Norway maple cotyledons, whereas in the sycamore cotyledons, equal amounts of reduced and oxidized Asc forms were detected at the 60–40% WC dehydration stages. Strikingly, a fivefold higher AsA/DHA ratio was detected in cotyledons dehydrated to 30% WC.

### 3.6. Redox Forms of MsrB2

**Figure 9:**
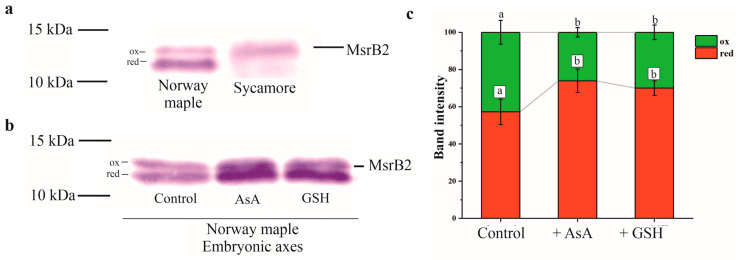
Characterization of the redox state of the MsrB2 protein. (**a**) Immunoblot analyses of MsrB2 proteins in two *Acer* species. Two bands corresponding to two redox states are visible only in Norway maple. (**b**) Immunoblot analyses of MsrB2 proteins using non-treated Norway maple protein extract (control) and protein extract incubated in vitro with the reducing reagents, ascorbic acid (AsA, lane 2) or GSH (lane 3). (**c**) Densitometric analysis of the oxidized and reduced forms of MsrB2. The data are the means of three independent replicates ± the STDs. The same letters indicate groups that are not significantly different according to Tukey’s test.

MsrB2 was visualized as two distinct bands solely in Norway maple seeds (Figure 9A). The lower and higher bands correspond to reduced and oxidized forms, respectively, as was shown by Vieira Dos Santos et al. [56]. These two bands could be distinguished by Western blotting in which a secondary antibody conjugated to AP and its substrate were used, which was not the major method used in this study because this method is less sensitive than chemiluminescence. The ratio between the two bands was investigated further to determine whether the two MsrB2 redox forms could be affected by AsA and GSH which are electron sources for the systems regenerating the activity for Msr enzymes. Following incubation of proteins extracted from hydrated Norway maple seeds (50% WC) with these reducing compounds, the lower band, corresponding to the reduced MsrB2 form was found to exhibit a 15% higher intensity than that noticed in the control assay (Figure 9B,C). This confirms that both AsA and GSH are likely involved in the reduction of the MsrB2 enzyme in hydrated Norway maple seeds. The effect of incubation with AsA or GSH was not observed in desiccated Norway maple seeds and in sycamore seeds regardless of dehydration stage (Figure S2).

### 3.7. Correlations

**Figure 10:**
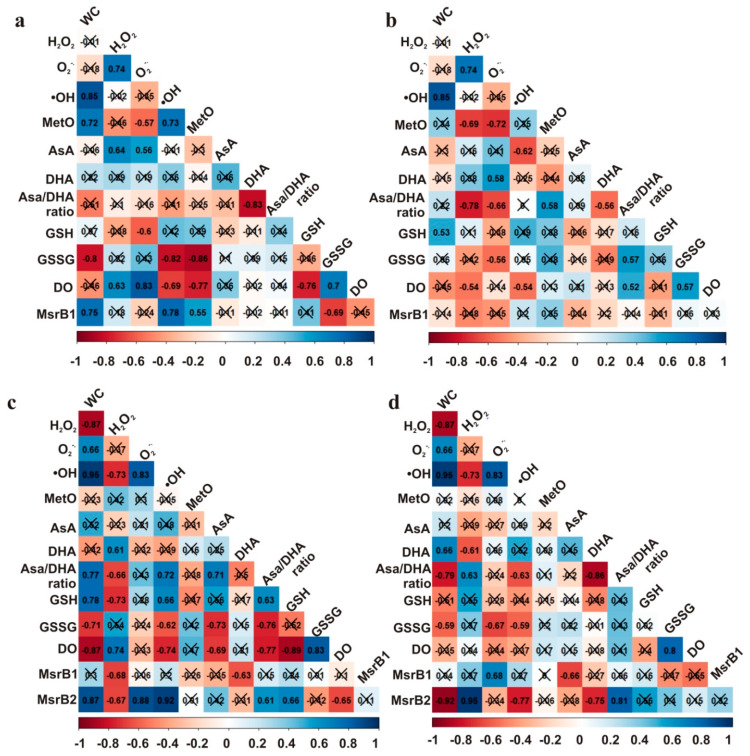
Correlation matrices for the (**a**) embryonic axes and (**b**) cotyledons of Norway maple and the (**c**) embryonic axes and (**d**) cotyledons of sycamore.

The Pearson correlation coefficient (R) was calculated between all measured parameters affecting redox status in seeds to investigate which of them is correlated to the MetO levels and MsrB2 abundance (Figure 10). Interestingly, MetO levels were positively correlated with •OH, and with the abundance of MsrB2 in the embryonic axes of only Norway maple seeds; of note, the levels of MetO and •OH were also linked to GSSG levels in these axes. In Norway maple cotyledons, MetO levels were negatively correlated with H_2_O_2_ and O_2_•^−^ levels and positively with the AsA/DHA ratio, which was linked to the content of the two ROS types (Figure 10B). No correlations were found for MetO levels in sycamore seeds (Figure 10C,D). The abundance of MsrB1 was not related to the loss of water, but instead was linked to H_2_O_2_ levels in the embryonic axes (Figure 10C) and O_2_•^−^ levels in the cotyledons (Figure 10D). The abundance of MsrB2 was correlated with changes in dehydration, ROS levels and the AsA/DHA ratio. Results presented in this report were also compared to NAD(P) concentrations and their redox status reported in Norway maple and sycamore seeds at identical dehydration and desiccation stages [51] and are shown in Table S1. MetO levels (Figure 2), when compared to NAD(P) concentrations and their redox status reported in Norway maple and sycamore seeds at identical dehydration and desiccation stages [51], were negatively correlated with NAD(P)H/NAD(P) ratios only in Norway maple seeds (Table S1). MetO levels were also correlated with NAD(P)H levels only in Norway maple seeds (Table S1), underlining that MetO levels, similarly to pyridine nucleotides [51], are involved in desiccation tolerance. Besides, MetO levels were correlated with the activity of NADH^−^ or NADPH-dependent reductases solely in Norway maple seeds (Table S1). To conclude, a clear correlation involving •OH level, peptide-bound MetO content and MsrB2 abundance was reported solely in embryonic axes of Norway maple seeds.

## 4. Discussion

### 4.1. Dynamics of MetO Levels in Contrasted Seeds in Relation with ROS

The accumulation of H_2_O_2_ to ranges of desiccation that cause oxidative stress predominantly contributes to the loss of viability of recalcitrant seeds [1,15,16]. In contrast, desiccation-tolerant seeds survive extreme desiccation and further rehydration by applying a set of mechanisms to avoid cellular damage [26,65]. In this context, homeostasis of ROS metabolism is essential [66]. Oxidative eustress is defined as the physiological ROS levels used in redox signaling; this contrasts with oxidative distress, which introduces oxidative damage [67,68]. Additionally, H_2_O_2_ effects are dose specific, and H_2_O_2_ concentrations ranging from 1 to 10 nM are assumed to be involved in signaling, whereas higher concentrations are perceived by plants as oxidative distress [68]. ROS homeostasis is more complex in recalcitrant seeds, because slightly increased ROS levels can activate antioxidant enzymes [69] to avoid stress in desiccation-sensitive tissues, while decreasing ROS levels can reduce desiccation-induced damage and improve the viability of recalcitrant seeds [70]. In this context, elevated H_2_O_2_ levels detected in sycamore seeds, particularly at the end of the dehydration process (Figure 1A) might be considered as oxidative distress as compared to Norway maple, where H_2_O_2_ level is eightfold lower. Thus, the ROS functions might differ in the two *Acer* species because of distinct dynamics of H_2_O_2_ levels during seed drying (Figure 1A). The amount of O_2_•^−^ in Norway maple and sycamore seeds (Figure 1B) is more complex to interpret, especially when previous studies have clearly shown that increased levels are present during *Acer* seed desiccation [42]; however, other studies claimed that desiccated seeds produce less ROS because only non-enzymatic reactions can generate them [15,24]. In general, the production of O_2_•^−^ can be used as a stress biomarker; however, it is not effective at distinguishing the different dehydration stages in recalcitrant seeds [71]. Similar to that which occurred for the H_2_O_2_ that accumulates, the excess •OH detected in sycamore seeds (Figure 1C) might be considered a harmful byproduct of oxidative metabolism; however, an increasing number of studies have demonstrated the regulatory and signaling roles of •OH [72].

Peptide-bound MetO contents were correlated with ROS levels only in Norway maple seeds (Figure 10). More specifically, levels of •OH were strongly reflected in MetO contents in the embryonic axes, whereas in the cotyledons, the levels of H_2_O_2_ and O_2_•^−^ were negatively linked to MetO levels. This particular relationship in the orthodox seeds (Figure 10) indicates a putative signaling role of this type of ROS. Most interestingly, among ROS, •OH is known to strongly contribute to Met oxidation [28]. The percentage of MetO in dried *Acer* seeds was in the 20 to 30% range (Figure 2). This observation is in line with the fact that desiccation-tolerant tissues experiencing intense changes in their cellular redox state [73]. The constant oxidation state of seeds [74] might explain the relatively high MetO levels reported in *Acer* seeds (Figure 2). Massive but selective protein oxidation via carbonylation has been reported to occur during important transitions in seeds, such as seed germination [75] and alleviation of seed dormancy [76]. Met oxidation levels differ in plant tissues and are modulated by environmental conditions. Unstressed leaves displayed MetO percentages reaching 2% in Arabidopsis [77] and 18% in pea [78]. However, photooxidative treatment increased MetO percentages up to 60% in Arabidopsis chloroplastic proteins [79]. The progressive decrease in WC affected the contents of peptide-bound MetO in developing seeds of Norway maple, whereas the MetO levels did not change in developing sycamore seeds [41]. In this context, changes in Met oxidation might be related to desiccation tolerance, which is lacking in sycamore seeds. Of note, vegetative tissues that lack desiccation tolerance, such as leaves subjected to water stress, displayed no change in protein-bound MetO levels [78]. Altogether, these data indicate that the MetO levels might be elevated by severe oxidative stress conditions. Consistently, an increased MetO level was thus noticed in the Arabidopsis catalase 2 mutant [31]. MetO levels, when compared to NAD(P) concentrations and their redox status, assumed as involved in desiccation tolerance in *Acer* seeds [51], displayed important correlations with Norway maple seeds (Table S1), underlining that MetO levels, similarly to pyridine nucleotides [51], are involved in desiccation tolerance.

### 4.2. Dynamics of MsrB Abundance

In this work, we focused on two MsrB proteins, MsrB1 and MsrB2, that have been reported to be essential actors in the preservation of seed longevity [39] and thus, studied regarding their potential roles in desiccated Norway maple and dehydrated sycamore seeds. Importantly, plastidial Msrs respond immediately to ROS accumulation to avoid oxidative stress [78]; however, dry mature seeds do not contain any photosynthetically active tissue [80]. In this context, in dry seeds, the role of MsrB1 and MsrB2 in MetO reduction goes far beyond the preservation of photosystem antennae proposed in Arabidopsis leaves [77]. Met oxidation caused by excessive ROS production is also considered a form of protection of protein structure and function during excessive ROS production [81]. Thus, the MetO percentage in Arabidopsis plants subjected to high light stress was twofold higher in a double mutant-lacking plastidial MsrB1 and MsrB2 isoforms than in WT [77].

The progressive decline in metabolism is unique to orthodox seeds [80]. Based on Western blot analysis, the abundance of MsrB2 in Norway maple embryonic axes significantly declined during the 30–10% WC desiccation stages (Figure 3). The lowest abundance of MsrB2 (Figure 4) together with the lowest level of MetO (Figure 2) during the final stages of desiccation, reflected redox-controlled deceleration of metabolism. Recalcitrant seeds do not undergo such a transition [5]; this is associated with a large increase in MsrB2 abundance in sycamore cotyledons during the 40–30% dehydration stages (Figure 4D). In general, MsrB2 was more abundant in sycamore embryonic axes (Figure 4C) than in the cotyledons (Figure 4D). Interestingly, a strong correlation between MsrB2 abundance and WC was reported; this correlation was positive in the embryonic axes (Figure 10C) but negative in the cotyledons, and the abundance of MsrB2 increased during the dehydration process in a way similar to H_2_O_2_ levels variation (Figure 10D). MsrB1 was detected only in sycamore seeds (Figure 3A,B); however, there was no correlation between the decrease in WC and the protein abundance of MsrB1 (Figure 10C,D). Importantly, such a relation was reported for the MsrB2 protein. The abundance of MsrB2 declined as desiccation progressed in the embryonic axes of Norway maple seeds (Figure 4A). In the cotyledons, the level of MsrB2 fluctuated (Figure 3B), and the protein was still detected in large amounts at the final desiccation stage. Hypothetically, we can speculate that MsrB2 might compensate for the absence of MsrB1, which was not detected in Norway maple, however this requires further studies.

MsrB2 was present only in the oxidized form in sycamore seeds, whereas in Norway maple seeds, the two redox forms were detected, with the reduced form being more abundant (Figure 9A, Figure S2). Additionally, the presence of AsA and GSH seemed to promote the reduced form of MsrB2 in Norway maple seeds (Figure 9B, Figure S2). The two reductants, AsA and GSH, were assumed to be potential candidates for the regeneration of oxidized Msrs in developing *Acer* seeds [41]. Since dithiotreitol was effective in Msrs reduction [59], it is plausible that several types of low-molecular-weight antioxidants donate protons and act in the reducing pathways for these enzymes, particularly during the enormous restriction of water during desiccation, that tremendously limits molecular mobility [18]. In desiccated seed tissues, relatively weak correlations between levels of AsA and GSH or MsrB2 were noticed in Norway maple seeds (Figure 10A,B) in contrast to those reported as strong in *Acer* developing seeds [41]. As such, a negative correlation between the GSSG level and the abundance of MsrB2 was reported in Norway maple seed embryonic axes (Figure 10A). Interestingly, in metabolically active sycamore seeds, the abundance of MsrB2 was correlated with the GSH level, DO and AsA/DHA ratio (Figure 10C,D).

Glutathione and Asc are crucial elements of the response to drought stress and desiccation; however, in sensitive tissues, these elements are less active [82]. GSH has long been considered a major antioxidant in desiccation tolerance [16]. In contrast, Asc has been less studied, particularly in woody plant species [51]. The back-and-forth mechanism for the regeneration of DHA to AsA is based on the enzymatic transition of GSH to the GSSG performed by dehydroascorbate reductase (DHAR) [83], which activity is distinct in orthodox and recalcitrant *Acer* seeds [42,84]. The embryonic axes of seeds of both *Acer* species accumulated predominantly DHA (Figure 6), confirming that DHA is the main Asc form in dry seeds, whereas glutathione occurs in equal amounts of reduced and oxidized forms [85]. In particular, the embryonic axes of sycamore seeds seem to display symptoms of a less effective Foyer–Halliwell–Asada cycle [86] because of the parallel accumulation of GSH (Figure 5) and DHA (Figure 7), distinct levels of redox couples of pyridine nucleotides and their redox state [51], and less active DHAR [42,84]. This enzyme might not be sufficiently preserved under water stress conditions, due to a lower abundance of protective proteins such as dehydrins and small heat shock proteins in recalcitrant *Acer* seeds [50].

### 4.3. Importance of the Msr System in Seed Physiology

The first report concerning Msr activity in plants was published in the sixties [87]. More studies concerning the Msr’s role have been published since then, and they are undoubtedly involved in protecting cells from biotic and abiotic stress [34], but many unknowns still need to be clarified, especially in the seed context. It seems likely that Msrs play a more complex role in plants than in yeast or mammals due to the much larger number of isoforms and the different types of cellular localization [27,35]. Initially, the localization of MrsB1 and MrsB2, which are plastidic isoforms, was limited mainly to photosynthetic tissues, because chloroplasts are major sites of ROS production [34,88]. The presence of the MrsB1 and MrsB2 enzymes has been confirmed in the majority of plant organs, including the flowers, stems, and roots, as well as the seeds, which suggests that MsrB1/B2 do not have a specific organ location and are present in various types of plastids [39,59]. Thus, they were detected in *Arabidopsis thaliana* and *Medicago truncatula* dry seeds [39] and recently, MsrB1 and MsrB2 were confirmed to be present in developing seeds of both Norway maple and sycamore [41]. There is evidence that MsrB2 in particular might be involved in the seed maturation process. Stolarska et al. [41] documented that MsrB2 was abundant during the final stages of maturation in both species of *Acer*. A relatively high amount of MsrB2 was also recorded during maturation of *Medicago trantacula* seeds. Such results were not observed for the second isoform, MsrB1, suggesting that mainly MsrB2 might participate in seed responses to drying and in improved storability [39]. The role of MsrB proteins has also been widely analysed in the case of biotic stress. In *Capsicum annuum* leaves, CaMsrB2 is involved in regulation of pathogen defence responses and oxidative stress [89]. The role of MsrB proteins in response to photooxidative stress was also investigated; in young leaves, there was no noticeable change in the amount of MsrB1/B2 protein, whereas in old leaves, a slight increase was noticed [59]. The involvement of MsrB proteins in the alleviation of water stress is uncertain. Vieira Dos Santos et al. [59] established that there was no noticeable change in the abundance of either MsrB1 or MsrB2 proteins in the leaves of Arabidopsis plants under water stress. However, rice plants overexpressing the *MsrB2* gene from pepper displayed enhanced tolerance to water deficiency conditions [90]. These discrepancies might originate from the different intensities of the stress applied in these studies. Here, we observed substantial changes in MsrB2 abundance particularly in orthodox seeds. We highlighted a positive correlation between •OH levels and the abundance of MsrB2 in the embryonic axes of only Norway maple seeds (Figure 10A), revealing a likely cause-and-effect sequence in redox regulation that is lacking in sycamore seeds. ROS-MetO-Msr interaction seems to be more intricate in sycamore seeds because elevated H_2_O_2_ levels (Figure 1A) might cause oxidative distress [67,68] and negatively affect the abundance of MrsB1 and MsrB2 predominantly in embryonic axes (Figure 10C).

When comparing *Acer* embryonic axes that display more intense redox changes than cotyledons, the abundance of MsrB2 (Figure 4) was positively correlated with the activity of NADPH-dependent reductases in Norway maple, whereas the abundance of MsrB1 was strongly negatively correlated with the activity of NADH-dependent reductases and strongly positively with the activity of NADPH-dependent reductases (Table S1). Of note, MsrB2 is also assumed to be involved in desiccation tolerance in developing Norway maple seeds [41]. Most importantly, MsrB1 and MsrB2 were previously reported to be essential for the longevity preservation in *Medicago* and Arabidopsis seeds [39]. Based on our data gained here in contrasted *Acer* seeds, we propose that MsrB2 participates in relation with the main redox determinants [51] in defining the desiccation tolerance in orthodox seeds. Taken together, these findings strongly support a pivotal role of MsrBs in seed physiology, particularly during the drying phase.

## 5. Conclusions

Desiccation tolerance is a strategy that allows survival during the severe dehydration of seeds. Differences in orthodoxy and recalcitrance in *Acer* seeds are still being elucidated, and Msrs are little-known players in terms of desiccation tolerance. Upon desiccation, Norway maple seeds accumulated less ROS as compared to sycamore seeds. A clear relationship between the levels of •OH and MetO as well as the abundance of MsrB2 was reported solely in Norway maple seeds. These oxidation-induced events might involve reversible posttranslational modifications that occur predominantly in orthodox seeds. Additionally, the reduced form of MsrB2 was predominant in Norway maple seeds indicating more active regenerating systems, that could include ascorbate and glutathione. In conclusion, Msrs, particularly the MsrB2 plastidial isoform, should be considered an important element allowing proper maintenance in protein redox status during the desiccation phase in orthodox seeds.

## Supplementary Materials

The following are available online at https://www.mdpi.com/2076-3921/9/5/391/s1, Figure S1: Histochemical detection of ROS, Figure S2: Redox status of MsrB2, Table S1: Correlation coefficient.
