# Supplementary material for: Peptide-Bound Methionine Sulfoxide (MetO) Levels and MsrB2 Abundance Are Differentially Regulated during the Desiccation Phase in Contrasted Acer Seeds
Source: Antioxidants (Basel). 2020 May 7;9(5):391. doi: 10.3390/antiox9050391 (PMC7278694; doi:10.3390/antiox9050391)

Figure S2. Redox status of MsrB2. The MsrB2 protein was investigated in terms of occurrence in redox and oxidized form in seed proteins of two *Acer* species, Norway maple and sycamore. Two bands corresponding to two redox states were visible only in hydrated Norway maple seeds (50% water content). The MsrB2 was continuously oxidized in sycamore seeds independently on dehydration stage. To distinguish reduced and oxidized form of MsrB2, proteins extracted from embryonic axes were incubated in vitro with the reducing reagents 1 mM ascorbic acid (abbreviated as A) or 1mM GSH (abbreviated as G). None treatment were applied to control samples (C).

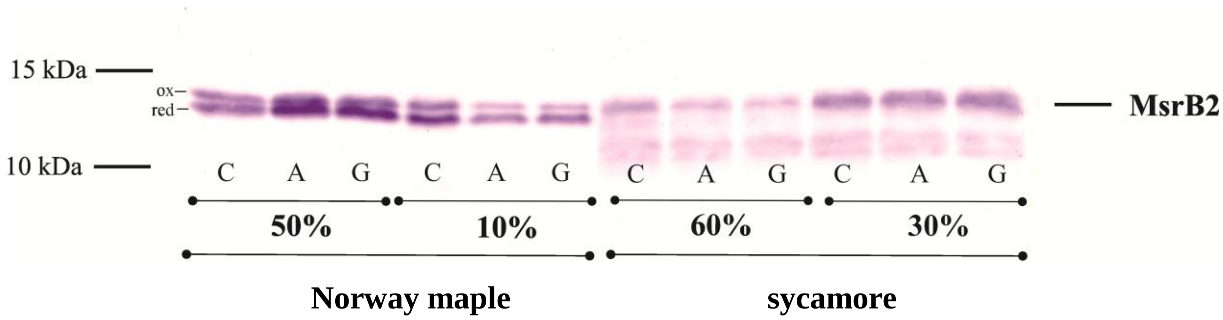

Supplement: Supplementary file 1 [file antioxidants-09-00391-s001.zip › Figure S2.pdf]
