# Supplementary material for: Peptide-Bound Methionine Sulfoxide (MetO) Levels and MsrB2 Abundance Are Differentially Regulated during the Desiccation Phase in Contrasted Acer Seeds
Source: Antioxidants (Basel). 2020 May 7;9(5):391. doi: 10.3390/antiox9050391 (PMC7278694; doi:10.3390/antiox9050391)
Supplement: Supplementary file 1 [file antioxidants-09-00391-s001.zip › Table S1.pdf]

Table S1. Correlation matrices calculated for embryonic axes (A) and cotyledons (B) of Norway maple (*Acer platanoides*), and embryonic axes (C) and cotyledons (D) of sycamore (*Acer pseudoplatanus*) based on levels of hydrogen peroxide (H_2O_2), superoxide anion radical ($O_2^{\bullet-}$), hydroxyl radical ($^{\cdot}OH$), ascorbic acid (AsA), dehydroascorbic acid (DHA), total pool of ascorbate (Asc), Asa to DHA ratio (AsA/DHA), levels of reduced glutathione (GSH), oxidized glutathione (GSSG), degree of oxidation of glutathione (DO), water content (WC), levels of protein-bound methionine sulfoxide (MetO), and the abundance of methionine sulfoxide reductase isoforms (MsrB1, MsrB2) against data published in Alipour et al. (2020) referring to levels of reduced (NADH) nicotinamide adenine dinucleotide (NAD), NADH to NAD^+ ratio ($NADH/NAD^+$ ratio), levels of reduced (NADPH) nicotinamide adenine dinucleotide phosphate (NADP), NADPH to $NADP^+$ ratio ($NADPH/NADP^+$ ratio) and reducing power based on NAD(P) concentrations. The intensity of color refers to the strength of correlation (R) coefficient (blue, positive; red negative). The intensity of red to orange color refers to the *P* value.

A) Norway maple embryonic axes

R	H_2O_2	$O_2^{\cdot-}$	$^{\cdot}OH$	AsA	DHA	AsA/DHA	GSSG	GSH	DO	WC	MsrB2	MetO
**NADH**	-0,7315	-0,7456	-0,0187	-0,5351	-0,2701	0,2535	-0,1204	0,5618	-0,6083	-0,0912	0,0451	0,2868
**$NADH/NAD^+$ ratio**	-0,2618	-0,4529	0,2369	0,1836	0,4533	-0,2376	-0,1341	0,4306	-0,5190	-0,1316	-0,1311	0,1824
**NADPH**	0,6400	0,8726	-0,4359	0,5786	0,2713	-0,3037	0,5024	-0,7957	0,8727	-0,2325	-0,4758	-0,6124
**$NADPH/NADP^+$ ratio**	0,5465	0,8032	-0,5277	0,2225	-0,2053	0,1408	0,5042	-0,5887	0,8740	-0,2433	-0,2095	-0,5828
**reducing power**	0,7277	0,8195	-0,1567	0,5742	0,1708	-0,1069	0,2438	-0,5272	0,6974	-0,1016	-0,1267	-0,3879
**NADH-dependent reductases**	0,3010	0,0501	0,4034	0,3719	0,7973	-0,6471	0,0074	-0,0219	-0,0293	0,1727	0,0097	0,0079
**NADPH-dependent reductases**	-0,4147	-0,4074	0,4636	-0,5202	-0,3280	-0,0759	-0,6663	0,0953	-0,4465	0,7498	0,6072	0,6851

P value	H_2O_2	$O_2^{\cdot-}$	$^{\cdot}OH$	AsA	DHA	AsA/DHA	GSSG	GSH	DO	WC	MsrB2	MetO
**NADH**	0,0019	0,0014	0,9471	0,0398	0,3303	0,3620	0,6690	0,0293	0,0161	0,7465	0,8732	0,3001
**$NADH/NAD^+$ ratio**	0,3459	0,0900	0,3953	0,5124	0,0897	0,3939	0,6338	0,1091	0,0474	0,6400	0,6414	0,5153
**NADPH**	0,0102	<,0001	0,1044	0,0238	0,3280	0,2712	0,0563	0,0004	<,0001	0,4044	0,0730	0,0152
**$NADPH/NADP^+$ ratio**	0,0351	0,0003	0,0432	0,4254	0,4629	0,6168	0,0553	0,0210	<,0001	0,3821	0,4537	0,0226
**reducing power**	0,0021	0,0002	0,5771	0,0252	0,5427	0,7045	0,3813	0,0434	0,0039	0,7185	0,6527	0,1531
**NADH-dependent reductases**	0,2757	0,8594	0,1359	0,1723	0,0004	0,0091	0,9791	0,9383	0,9176	0,5381	0,9726	0,9776
**NADPH-dependent reductases**	0,1243	0,1318	0,0817	0,0468	0,2327	0,7880	0,0067	0,7354	0,0952	0,0013	0,0164	0,0048

B) Norway maple cotyledons

R	H_2O_2	$O_2^{\cdot -}$	$^{\cdot}OH$	AsA	DHA	AsA/DHA	GSSG	GSH	DO	WC	MsrB2	MetO
**NADH**	-0,7728	-0,6246	0,3010	-0,0760	-0,3584	0,8888	0,4801	0,2374	0,3543	0,5074	0,0268	0,6359
**$NADH/NAD^+$ ratio**	0,9081	0,7231	0,0678	-0,0869	0,4904	-0,9045	-0,5021	0,0045	-0,5614	-0,0288	-0,2094	-0,6775
**NADPH**	-0,7004	-0,6752	0,3656	-0,1730	-0,4267	0,7711	0,5774	0,2136	0,2446	0,5090	0,2735	0,5973
**$NADPH/NADP^+$ ratio**	0,6669	0,7931	-0,5814	0,5937	0,5734	-0,5085	-0,5028	-0,2277	-0,0888	-0,4087	-0,5704	-0,6159
**reducing power**	0,4428	0,5934	-0,4743	0,5975	0,5250	-0,2659	-0,4828	-0,1952	-0,0303	-0,2628	-0,5760	-0,4602
**NADH-dependent reductases**	-0,7706	-0,7420	0,4023	-0,3232	-0,3757	0,8374	0,4853	0,3589	0,2731	0,5060	0,0381	0,6742
**NADPH-dependent reductases**	-0,6759	-0,7555	0,0447	-0,5415	-0,3607	0,3373	0,3905	0,0125	0,3589	-0,3011	0,5873	0,4848

***P* value**	H_2O_2	$O_2^{\cdot -}$	$^{\cdot}OH$	AsA	DHA	AsA/DHA	GSSG	GSH	DO	WC	MsrB2	MetO
**NADH**	0,0007	0,0128	0,2756	0,7878	0,1897	<,0001	0,0701	0,3942	0,1951	0,0535	0,9246	0,0108
**$NADH/NAD^+$ ratio**	<,0001	0,0023	0,8104	0,7582	0,0635	<,0001	0,0565	0,9872	0,0294	0,9189	0,4539	0,0055
**NADPH**	0,0036	0,0057	0,1802	0,5375	0,1127	0,0008	0,0242	0,4446	0,3796	0,0526	0,3240	0,0187
**$NADPH/NADP^+$ ratio**	0,0066	0,0004	0,0230	0,0196	0,0254	0,0529	0,0561	0,4145	0,7529	0,1304	0,0264	0,0145
**reducing power**	0,0984	0,0197	0,0741	0,0187	0,0445	0,3382	0,0683	0,4857	0,9145	0,3439	0,0246	0,0844
**NADH-dependent reductases**	0,0008	0,0015	0,1371	0,2399	0,1676	<,0001	0,0667	0,1890	0,3247	0,0543	0,8928	0,0058
**NADPH-dependent reductases**	0,0057	0,0011	0,8743	0,0371	0,1866	0,2189	0,1501	0,9647	0,1890	0,2754	0,0213	0,0670

C) Sycamore embryonic axes

R	H_2O_2	$O_2^{\cdot -}$	$^{\cdot}OH$	AsA	DHA	AsA/DHA	GSSG	GSH	DO	WC	MsrB1	MsrB2	MetO
**NADH**	-0,9072	0,4580	0,8227	0,4211	-0,4147	0,6767	-0,6141	0,7410	-0,7755	0,9154	0,5444	0,7249	0,0194
**$NADH/NAD^+$ ratio**	0,7410	-0,3401	-0,7061	-0,5846	0,2806	-0,7230	0,7300	-0,7935	0,9212	-0,8607	-0,1444	-0,6347	0,0899
**NADPH**	-0,5793	0,6877	0,8336	0,3503	-0,3045	0,5354	-0,5374	0,5476	-0,6470	0,7980	0,0595	0,7267	0,1012
**$NADPH/NADP^+$ ratio**	0,6374	-0,4813	-0,7289	-0,5262	0,2686	-0,6621	0,6654	-0,6501	0,8031	-0,8219	0,0022	-0,6647	-0,0182
**reducing power**	0,7678	-0,4421	-0,7694	-0,5745	0,3048	-0,7327	0,7350	-0,7663	0,9016	-0,9006	-0,1293	-0,7063	0,0596
**NADH-dependent reductases**	0,8327	-0,3615	-0,5360	0,1880	0,6588	-0,3131	0,1321	-0,3613	0,2831	-0,5958	-0,8680	-0,5131	0,0007
**NADPH-dependent reductases**	-0,8659	0,2134	0,5229	-0,0485	-0,6364	0,4195	-0,2541	0,4958	-0,4012	0,6155	0,9213	0,4342	-0,1496

***P* value**	H_2O_2	$O_2^{\cdot -}$	$^{\cdot}OH$	AsA	DHA	AsA/DHA	GSSG	GSH	DO	WC	MsrB1	MsrB2	MetO
**NADH**	<,0001	0,1343	0,0010	0,1728	0,1801	0,0157	0,0336	0,0058	0,0030	<,0001	0,0673	0,0076	0,9523
**$NADH/NAD^+$ ratio**	0,0058	0,2794	0,0103	0,0459	0,3771	0,0079	0,0070	0,0021	<,0001	0,0003	0,6542	0,0266	0,7812
**NADPH**	0,0484	0,0134	0,0008	0,2643	0,3359	0,0728	0,0716	0,0654	0,0230	0,0019	0,8543	0,0074	0,7543
**$NADPH/NADP^+$ ratio**	0,0258	0,1132	0,0072	0,0789	0,3987	0,0190	0,0182	0,0221	0,0017	0,0010	0,9946	0,0184	0,9552
**reducing power**	0,0035	0,1501	0,0034	0,0507	0,3353	0,0067	0,0065	0,0037	<,0001	<,0001	0,6888	0,0102	0,8540
**NADH-dependent reductases**	0,0008	0,2483	0,0725	0,5584	0,0198	0,3217	0,6824	0,2485	0,3725	0,0409	0,0003	0,0880	0,9983
**NADPH-dependent reductases**	0,0003	0,5054	0,0811	0,8810	0,0261	0,1746	0,4256	0,1012	0,1962	0,0331	<,0001	0,1584	0,6426

D) Sycamore cotyledons

**R**	H_2O_2	$O_2^{\cdot -}$	$^{\cdot}OH$	AsA	DHA	AsA/DHA	GSSG	GSH	DO	WC	MsrB1	MsrB2	MetO
**NADH**	-0,4909	0,9454	0,8884	-0,1757	0,1824	-0,3059	-0,6444	-0,2609	-0,4141	0,7452	0,5531	-0,4670	-0,3782
**$NADH/NAD^+$ ratio**	0,5953	-0,8121	-0,8914	0,0532	-0,3703	0,4673	0,6080	0,1226	0,4909	-0,8029	-0,4088	0,5883	0,3516
**NADPH**	-0,7683	0,5625	0,7616	0,2523	0,4162	-0,3133	-0,2361	-0,5592	0,0887	0,7436	-0,0999	-0,6977	-0,0486
**$NADPH/NADP^+$ ratio**	0,5363	-0,6952	-0,6211	0,1388	0,1521	-0,0376	0,5080	0,2052	0,0672	-0,5257	-0,2007	0,3708	0,3257
**reducing power**	0,5192	-0,7536	-0,6507	0,1572	0,1143	0,0135	0,5975	0,1077	0,2261	-0,5492	-0,3154	0,3613	0,3303
**NADH-dependent reductases**	-0,3554	0,9441	0,8002	-0,2489	-0,0140	-0,1513	-0,6553	-0,2160	-0,3941	0,6275	0,6114	-0,3253	-0,3940
**NADPH-dependent reductases**	0,7096	-0,7324	-0,9492	-0,0298	-0,6529	0,8219	0,6449	0,4484	0,4913	-0,9568	-0,2770	0,8079	0,4452

***P* value**	H_2O_2	$O_2^{\cdot -}$	$^{\cdot}OH$	AsA	DHA	AsA/DHA	GSSG	GSH	DO	WC	MsrB1	MsrB2	MetO
**NADH**	0,1051	<,0001	0,0001	0,5850	0,5705	0,3335	0,0237	0,4128	0,1808	0,0054	0,0622	0,1259	0,2254
**$NADH/NAD^+$ ratio**	0,0411	0,0013	<,0001	0,8695	0,2361	0,1256	0,0360	0,7042	0,1051	0,0017	0,1870	0,0442	0,2624
**NADPH**	0,0035	0,0569	0,0040	0,4289	0,1784	0,3214	0,4600	0,0587	0,7841	0,0056	0,7574	0,0116	0,8808
**$NADPH/NADP^+$ ratio**	0,0722	0,0121	0,0311	0,6671	0,6371	0,9077	0,0917	0,5224	0,8356	0,0792	0,5317	0,2354	0,3016
**reducing power**	0,0836	0,0047	0,0219	0,6256	0,7236	0,9667	0,0402	0,7390	0,4799	0,0644	0,3180	0,2486	0,2944
**NADH-dependent reductases**	0,2568	<,0001	0,0018	0,4354	0,9656	0,6388	0,0207	0,5002	0,2049	0,0289	0,0346	0,3021	0,2050
**NADPH-dependent reductases**	0,0097	0,0068	<,0001	0,9269	0,0213	0,0010	0,0235	0,1437	0,1048	<,0001	0,3834	0,0015	0,1470
