# Supplementary material for: Peptide-Bound Methionine Sulfoxide (MetO) Levels and MsrB2 Abundance Are Differentially Regulated during the Desiccation Phase in Contrasted Acer Seeds
Source: Antioxidants (Basel). 2020 May 7;9(5):391. doi: 10.3390/antiox9050391 (PMC7278694; doi:10.3390/antiox9050391)

Figure S1. Histochemical detection of reactive oxygen species in dried *Acer* seeds. Hydrogen peroxide (H_2O_2) was visualized in whole Norway maple seeds and sycamore seeds as a reddish-brown stain formed by the reaction with DAB solution according to the method of Daudi and O'Brien (2012). Superoxide anion ($O_2^{\bullet-}$) was visualized as a dark blue-to-black stain formed by NBT using according to the protocol of Kumar et al. (2014). Images were taken on a plain white background using a Nikon D3100 digital camera attached to a binocular microscope.

Norway maple seeds

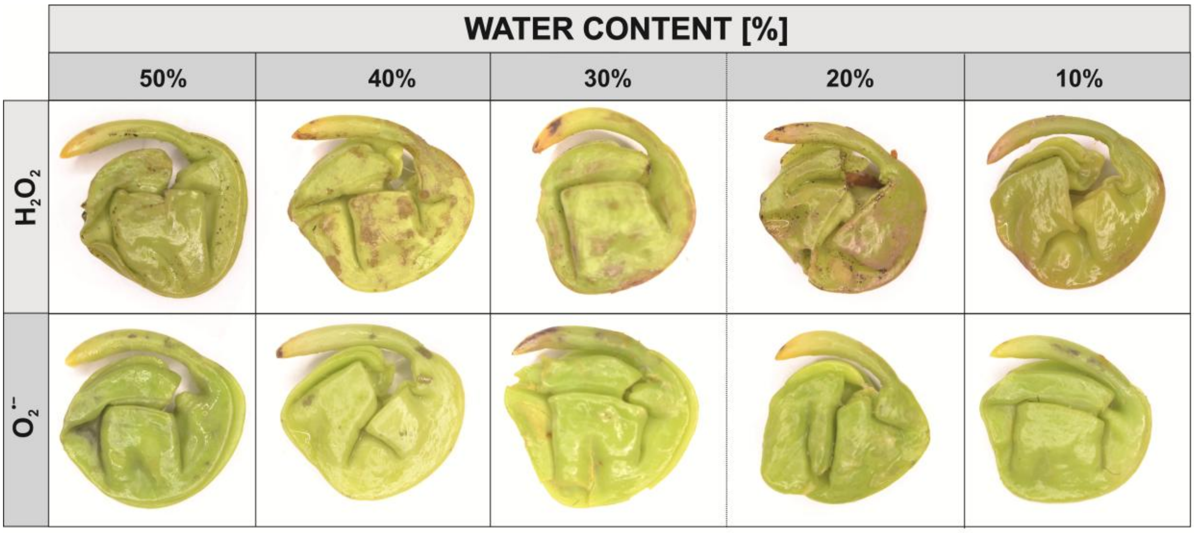

Sycamore seeds

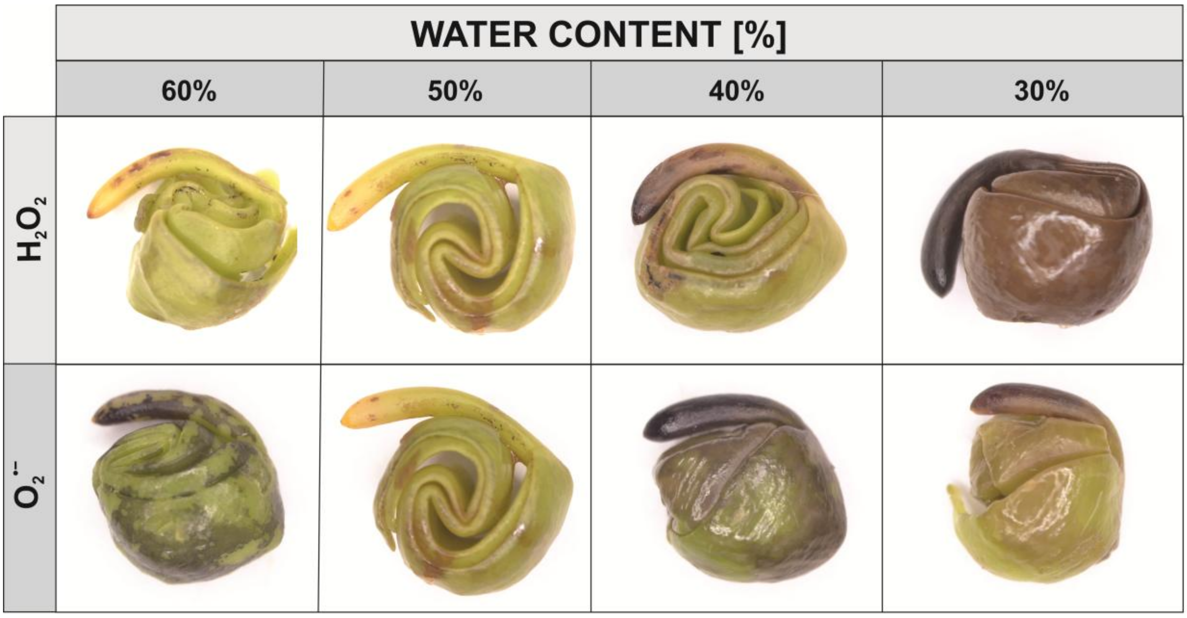

Supplement: Supplementary file 1 [file antioxidants-09-00391-s001.zip › Figure S1.pdf]
